# Enhanced genome assembly and a new official gene set for *Tribolium castaneum*

**DOI:** 10.1186/s12864-019-6394-6

**Published:** 2020-01-14

**Authors:** Nicolae Herndon, Jennifer Shelton, Lizzy Gerischer, Panos Ioannidis, Maria Ninova, Jürgen Dönitz, Robert M. Waterhouse, Chun Liang, Carsten Damm, Janna Siemanowski, Peter Kitzmann, Julia Ulrich, Stefan Dippel, Georg Oberhofer, Yonggang Hu, Jonas Schwirz, Magdalena Schacht, Sabrina Lehmann, Alice Montino, Nico Posnien, Daniela Gurska, Thorsten Horn, Jan Seibert, Iris M. Vargas Jentzsch, Kristen A. Panfilio, Jianwei Li, Ernst A. Wimmer, Dominik Stappert, Siegfried Roth, Reinhard Schröder, Yoonseong Park, Michael Schoppmeier, Ho-Ryun Chung, Martin Klingler, Sebastian Kittelmann, Markus Friedrich, Rui Chen, Boran Altincicek, Andreas Vilcinskas, Evgeny Zdobnov, Sam Griffiths-Jones, Matthew Ronshaugen, Mario Stanke, Sue J. Brown, Gregor Bucher

**Affiliations:** 1 Department of Computer Science, East Carolina University, Greenville, NC 27858 USA; 2 Division of Biology, Kansas State University, Manhattan, KS 66506 USA; 3 Institut für Mathematik und Informatik, Universität Greifswald, Greifswald, Germany; 4 Department of Genetic Medicine and Development, University of Geneva Medical School and Swiss Institute of Bioinformatics, 1211 Geneva, Switzerland; 5 Faculty of Biology, Medicine and Health, University of Manchester, Michael Smith Building, Oxford Road, Manchester, M13 9PT UK; 6 Department of Evolutionary Developmental Genetics, GZMB, University of Göttingen, Justus-von-Liebig-Weg 11, 37077 Göttingen, Germany; 7 Department of Ecology and Evolution, University of Lausanne and Swiss Institute of Bioinformatics, 1015 Lausanne, Switzerland; 8 Department of Biology, Miami University, Oxford, OH 45056 USA; 9 Institut für Informatik, Fakultät für Mathematik und Informatik, Georg-August-Universität Göttingen, Goldschmidtstr. 7, 37077 Göttingen, Germany; 10 Göttinger Graduiertenschule fur Neurowissenschaften Biophysik und Molekulare Biowissenschaften, Georg-August-Universität Göttingen, Göttingen, Germany; 11 Department of Developmental Biology, GZMB, University of Göttingen, Justus-von-Liebig-Weg 11, 37077 Göttingen, Germany; 12 Institute for Zoology: Developmental Biology, University of Cologne, Zülpicher Str. 47b, 50674 Cologne, Germany; 13 School of Life Sciences, University of Warwick, Gibbet Hill Campus, Coventry, CV4 7AL UK; 14 Department Developmental Biology, GZMB, University of Göttingen, Justus-von-Liebig-Weg 11, 37077 Göttingen, Germany; 15 Department of Developmental Biology, University of Göttingen, Justus-von-Liebig-Weg 11, 37077 Göttingen, Germany; 16 Institute of Zoology: Developmental Biology, University of Cologne, Zülpicher Weg 47b, 50674 Cologne, Germany; 17 Institut für Biowissenschaften, Universität Rostock, Albert-Einstein-Str. 3, 18059 Rostock, Germany; 18 Department of Entomology, Kansas State University, Manhattan, KS 66506 USA; 19 Department of Biology, Divison of Developmental Biology, Friedrich-Alexander-University of Erlangen-Nürnberg, Staudtstr. 5, 91058 Erlangen, Germany; 20 Department of Computational Molecular Biology, Max-Planck-Institute for Molecular Genetics, Ihnenstraße 63-73, 14195 Berlin, Germany; 21 Department of Biology, Division of Developmental Biology, Friedrich-Alexander-University of Erlangen-Nürnberg, Staudtstr. 5, 91058 Erlangen, Germany; 22 Oxford Brookes University, Centre for Functional Genomics, Gipsy Lane, Oxford, OX3 0BP UK; 23 Department of Anatomy and Cell Biology, Wayne State University, Detroit, MI 48202 USA; 24 Baylor College of Medicine, Houston, Texas USA; 25 Institute of Crop Science and Resource Conservation (INRES-Phytomedicine), Rheinische Friedrich-Wilhelms-University of Bonn, Bonn, Germany; 26 Institute for Insect Biotechnology, Justus-Liebig University of Giessen, Heinrich-Buff-Ring 26-32, 35392 Giessen, Germany; 27 Georg-August-Universität Göttingen, Göttingen, Germany

**Keywords:** *Tribolium castaneum*, Genome, Genome assembly Tcas5.2, Reannotation, Gene prediction, Gene set OGS3, RefSeq genome, Gene annotation, microRNA, miRNA

## Abstract

**Background:**

The red flour beetle *Tribolium castaneum* has emerged as an important model organism for the study of gene function in development and physiology, for ecological and evolutionary genomics, for pest control and a plethora of other topics. RNA interference (RNAi), transgenesis and genome editing are well established and the resources for genome-wide RNAi screening have become available in this model. All these techniques depend on a high quality genome assembly and precise gene models. However, the first version of the genome assembly was generated by Sanger sequencing, and with a small set of RNA sequence data limiting annotation quality.

**Results:**

Here, we present an improved genome assembly (Tcas5.2) and an enhanced genome annotation resulting in a new official gene set (OGS3) for *Tribolium castaneum*, which significantly increase the quality of the genomic resources. By adding large-distance jumping library DNA sequencing to join scaffolds and fill small gaps, the gaps in the genome assembly were reduced and the N50 increased to 4753kbp. The precision of the gene models was enhanced by the use of a large body of RNA-Seq reads of different life history stages and tissue types, leading to the discovery of 1452 novel gene sequences. We also added new features such as alternative splicing, well defined UTRs and microRNA target predictions. For quality control, 399 gene models were evaluated by manual inspection. The current gene set was submitted to Genbank and accepted as a RefSeq genome by NCBI.

**Conclusions:**

The new genome assembly (Tcas5.2) and the official gene set (OGS3) provide enhanced genomic resources for genetic work in *Tribolium castaneum*. The much improved information on transcription start sites supports transgenic and gene editing approaches. Further, novel types of information such as splice variants and microRNA target genes open additional possibilities for analysis.

## Background

The red flour beetle *Tribolium castaneum* is an excellent insect model system for functional genetics. In many respects the biology of *Tribolium* is more representative of insects than that of the fly *Drosophila melanogaster* [1–3]. This is especially true with respect to embryonic development: The *Tribolium* embryo is enveloped by extraembryonic membranes like most insects [4], develops embryonic legs, displays an everted head [5] and its posterior segments are formed sequentially from a posterior segment addition zone [6, 7]. With respect to postembryonic development, the *Tribolium* larval epidermal cells build most of the adult epidermis while in *Drosophila* they are replaced by imaginal cells [8]. In the telotrophic ovary type of *Tribolium* the biology of somatic stem cells can be studied independent of germline stem cells, which cease to divide prior to hatching [9]. *Tribolium* is also studied with respect to beetle specific evolutionary novelties such as elytra [10] and gin traps [11]. It is also amenable to studies of physiology such as the formation of the extremely hard cuticle [12], and the function of the cryptonephridial system [13], which is a model for unique adaptation to dry habitats. Odoriferous glands are studied to understand the production of toxic secretions without harming the animal [14]. Finally, *Tribolium* is a representative of the Coleoptera, which is the most species-rich taxon on earth [15] including many economically important pests such as leaf and snout beetles. Hence, it has been used as a model for pest control [16, 17]. In summary, *Tribolium* is useful for evolutionary comparisons of gene function among insects, for studying processes that are not represented in *Drosophila* and for pest control studies.

Research on gene function in *Tribolium* is fostered by an extensive toolkit. Transposon-mediated transgenesis has led to the development of imaging and misexpression tools, and has facilitated a large-scale insertional mutagenesis screen [18–24]. However, the main strength of the model system lies in its reverse genetics via RNAi. First, the RNAi response is very strong, reaching the null phenotype in those cases where a genetic mutant was available for comparison [25–28]. In addition, RNAi is environmental, i.e. cells very efficiently take up dsRNA from the hemolymph and the RNAi effect is transmitted from injected mothers to their offspring [29–31]. Based on this strength, a genome wide RNAi screen was performed (iBeetle screen), in which embryonic and other phenotypes were documented and made available via the iBeetle-Base [32–34]. Importantly, the genome wide collection of templates generated by iBeetle can be used for future screens directed at other processes. Recently, CRISPR/Cas9 mediated genome editing has been shown to work efficiently [35, 36].

An essential requirement for studying gene function is a high quality genome assembly and a well annotated gene set. Indeed, the first genome assembly, published in 2008 community database [37, 38] contributed significantly to the growth of the community and increased the diversity of research topics studied in *Tribolium*. However, in the first published *Tribolium* genome assembly a substantial number of scaffolds had not been anchored to any Linkage Group. Further, the first gene annotations were mainly based on the detection of sequence features by bioinformatics tools and homology to *Drosophila* genes and very few gene predictions were supported by RNA data. Hence, precision in the coding regions was limited, non-coding UTR sequences and transcription start sites were usually not defined and splice variants were not predicted.

Here, we made use of new sequencing and mapping techniques in order to significantly enhance the genomic resources of *Tribolium*. In the new *Tribolium* assembly, Tcas5.2, scaffold length has been increased fivefold (scaffold N50: 4753kbp). With the inclusion of RNA-Seq data, the precision of gene models was improved and additional features such as UTRs and alternative splice variants were added to 1335 gene models. 1452 newly predicted genes replaced a similar number of short genes that had been falsely predicted. The current set of gene models (OGS3) is the first NCBI RefSeq annotation for *Tribolium castaneum*. Based on the enhanced annotation we compared the degree of conservation of protein sequences between a number of model systems revealing *Tribolium* sequences appear less diverged compared to other Ecdysozoa. Moreover, with the identification of UTRs, we were able to map, for the first time in a beetle, potential target genes of the microRNA complement and identified a conserved target gene set for a conserved microRNA.

## Results

### Improving the scaffolding of the Tcas genome assembly

The first published *Tribolium* genome sequence (NCBI Tcas3.0) was based on a Sanger 7x draft assembly [38] totaling 160 Mb, 90% of which was anchored to pseudomolecules or Linkage Groups (LGs) representing linkage groups in the molecular recombination map [39]. However, several large scaffolds (up to 1.17 Mb) were not included. To improve this draft assembly, we sequenced the paired ends of three large-insert jumping libraries (appr. 3200 bp, 6800 bp, and 34,800 bp inserts, respectively). These sequences were used to link scaffolds in the Sanger assembly and fill small gaps. Further, whole genome physical maps produced from images of ultra-long individual molecules of *Tribolium* DNA labeled at restriction sites (BioNano Genomics) were used to validate the assembly and merge scaffolds. The entire workflow and key steps are described below.

Using the long-insert jumping libraries, Atlas-Link (Baylor College of Medicine; www.hgsc.bcm.edu/software/atlas-link) joined neighboring anchored scaffolds and added several unplaced scaffolds, reducing the total number of scaffolds from 2320 to 2236. Of these, three were manually split because the joined scaffolds were known to be on different linkage groups based on the molecular genetic recombination map, leading to a total of 2240 scaffolds. This analysis added formerly unplaced scaffolds to all LGs except LG4. In addition, 16 unplaced scaffolds were linked together.

We also took advantage of the new Illumina sequence information gained from the long insert jumping libraries to fill small gaps and extend contigs. GapFiller [40] added 77,556 nucleotides and closed 2232 gaps (Table 1). Specifically, the number of gaps of assigned length 50, which actually included gaps less than 50 nucleotides long or potentially overlapping contigs, was reduced by 65.6% (from 1793 to 615).

**Table 1:** Ungapped length and spanned gaps before and after running GapFiller

Molecule	Ungapped length before	Spanned gaps before	Ungapped length after	Spanned gaps after
LG1 = X	7,071,107	301	7,096,881	201
LG2	14,229,660	359	14,306,202	192
LG3	28,072,007	1451	28,315,770	929
LG4	11,540,046	300	11,632,658	160
LG5	14,111,830	358	14,196,565	193
LG6	8,262,430	555	8,332,882	407
LG7	15,084,119	429	15,185,902	258
LG8	12,870,760	577	12,987,347	378
LG9	14,900,846	634	15,007,071	384
LG10	7,070,154	498	7,128,489	365
Unplaced multi-contig	14,079,574	1111	14,205,681	874
Unplaced single-contig	4,020,722	–	4,021,060	–
Total	151,313,255	6573	152,416,508	4341

Finally, BioNano Genomics consensus maps were used to validate and further improve the assembly (for details, see [41]). More than 81% of Tcas5.2 was directly validated by alignment with BioNano Genomics Consensus maps, the number of scaffolds was reduced by 4% to 2148, and the N50 increased 3-fold to 4753.0 kb. In total, the N50 was increased almost 5-fold where superscaffolding with BioNano Genomics optical maps improved the contiguity of the assembly the most. Table 2 shows the extent to which each step of the workflow impacted the quality of the genome assembly.

**Table 2:** Assembly improvement

Assembly	Length	Scaffolds	Scaffold N50 (kbp)
Tcas 3.0	160,445,652	2320	976.4
After Atlas-Link	160,667,144	2240	1175.4
After GapFiller	160,744,700	2240	1176.7
After BioNano Genomics / Tcas 5.2	165,921,904	2148	4753.0

### Re-annotation of the *Tribolium* genome assembly

Re-annotation was performed using the gene finder AUGUSTUS [42]. For the current release, new data were available and incorporated as extrinsic evidence including RNA-Seq, ESTs (Expressed Sequence Tags) and protein sequences. The most impactful new information was the extensive RNA-Seq data (approximately 6.66 billion reads) covering different life stages and tissues. This allowed us to determine UTRs and alternative splice variants, which were not annotated in the previous official gene set. This increased both transcript coverage (Table 3) and the accuracy of the predicted gene features. The parameters of automated annotation were adjusted based on manual quality control of more than 500 annotations of previously published genes. The new gene set, OGS3, consists of 16,593 genes with a total of 18,536 transcripts. 15,258 (92%) genes have one isoform, 944 (5.7%) genes have two, 270 (1.6%) have three and 121 (0.7%) genes have more than three isoforms. During the re-annotation of the *Tribolium* gene set a basic parameter set for AUGUSTUS was developed and is now delivered with AUGUSTUS as parameter set “tribolium2012” (link for download: see Materials and Methods).

**Table 3:** Read alignments to OGS2 and OGS3 transcript sets. The numbers of alignments are shown. Only the best alignment(s) for each read are reported. The last row suggests that OGS2 may have a slight bias towards highly expressed genes

	OGS2	OGS3
Total number of alignments	4,634,356,882	7,418,675,525
Number of alignments per transcript	278,926	400,317
Number of aligned reads per exon position	285.77	260.45

### Major changes in the OGS3

We compared the previous official gene set OGS2 [37], which was ‘lifted’ to the new assembly, Tcas5.2, with the new OGS3 and found that 9294 genes have identical protein sequences, while 3039 genes have almost identical protein sequences (95% minimum identity and 95% minimum coverage). 1452 genes were completely new, meaning that they did not overlap any lifted OGS2 gene above the given thresholds. A similar number (1420) of predicted genes from OGS2 do not exist anymore in OGS3. We further analyzed the “lost” and “new” genes and found that our procedure was efficient in removing false positive annotations and in detecting novel true genes. First, based on the lack of a BLAST hit in invertebrates (e-value cutoff: e-05), GO annotation or RNA-Seq coverage we assume that the “lost” OGS2 annotations had been falsely annotated. Second, when examining the newly found genes, we observe that 528 of 1452 (36%) genes had significant BLAST hits in other insect species. Further, 690 of 997 (69.2%) of the new genes have at least one intron supported by RNA-Seq. New single exon genes have an average read coverage of about 550,000 reads per gene with minimum coverage of 11 reads per gene. The percentage of missing BUSCO genes was reduced from 0.7 to 0.4%. Together, these metrics indicate that real genes were newly annotated. Table 4 compares important characteristics between the previous and the current OGS.

**Table 4:** Annotation improvement

	OGS2	OGS3
Number of genes	16,561	16,593
Average coding length	1341 bp	1473 bp
Number of coding exons per transcript	4.32	5.02
GC content	0.4597%	0.4625%
Fraction of single exon genes	17.66%	17.74%
Number of introns (excluding UTR)	54,909 (54875)	63,211 (58837)
Fraction of RNA-Seq-supported introns	76.3%	86.2%
Average intron length	1167 bp	1362 bp

We further examined gene structure changes (not including the identification of splice variants). For this, we counted both, gene *join* and *split* events that occurred in the new gene set. *Joins* are indicated when the CDS of an OGS3 gene overlaped the CDSs of two or more genes from the previous gene set on the same strand. In total, we observe 949 such *join* events. In 485 (51%) of these events, the new intron of an OGS3 gene was supported by spliced read alignments spanning the gap between two neighboring OGS2 genes, suggesting that the annotations had erroneously been split in the previous annotation. We detected gene *split* events by counting gene *join* events where an old OGS2 gene joined multiple OGS3 genes. We observed 424 such events. In 45 cases (10%) the joining OGS2 intron had RNA-Seq support. Taken together, while > 50% of the joined genes were supported by sequencing data only 10% of the split events turned out to be likely false positives. This indicated that the parameter set was adequate to enrich for true annotations in the new gene set.

### RNA-Seq support for the gene sets

Analysis of differential gene expression has become an essential tool in studying the genetic basis of biological processes. Such analyses profit from a better gene model where a higher number of reads can be mapped. To test whether the new gene set performed better in such analyses, we mapped our collection of RNA-Seq reads to both (Table 3). In this analysis 6.66 billion RNA-Seq reads from *Tribolium* where mapped against the two gene sets (transcriptome) OGS3 and, for comparison, OGS2 with the alignment tool BLAT [43]. Alignments with less than 90% identity were discarded and only the best alignment was kept for each read. About 70% of the reads mapped to OGS2 whereas 81% mapped to OGS3.

To evaluate the splice sites in the new gene set we compiled a set of splices suggested by gaps in RNA-Seq read alignments compared to the genomic sequence (intron candidates). These RNA-Seq read alignments where filtered by a range of criteria (see Methods). In total this set contained 65,274 intron candidates. We refer to the term *multiplicity* of an intron candidate as the number of reads that were found to cross a given exon-exon boundary at the identical position. Some candidate introns are likely not introns of coding genes, e.g. from alignment errors or from spliced noncoding genes. Overall, candidate introns had an average multiplicity of 7898. 1403 candidate introns had a multiplicity of one while 3362 had a multiplicity smaller or equal to five. OGS3 contains about 30% more RNA-Seq supported introns than OGS2: 41,921 out of 54,909 introns in OGS2 (76.3%) and 54,513 out of 63,211 in OGS3 (86.2%) are identical to an intron suggested by RNA-Seq spliced read alignments (Table 4).

### BUSCO analysis reveals very high accuracy of the gene set

The completeness of OGS3 was assessed using BUSCO (Benchmarking Universal Single-Copy Orthologs) and compared to the value for OGS2 [44] and to those of other sequenced genomes [45–47]. The genome of *Drosophila melanogaster* can be assumed to be the best annotated genome of insects, the genome of *Apis mellifera* was recently re-annotated and is therefore comparable to the OGS3 from *Tribolium* and for *Parasteatoda tepidariorum*, for which the first genome version was just published with the peculiarity of large duplication events. Nearly all of the conserved genes from the BUSCO Arthropoda set where found in OGS2 and OGS3 (Table 5). OGS3 (99.6%) scored slightly better than OGS2 (99.3%). The completeness of OGS3 rivals that of *Drosophila* (99.8%) and is better than *Apis* (97.9%) or *Parasteatoda* (94.4%) (Table 5).

**Table 5:** BUSCO analysis

	Tcas OGS2	Tcas OGS3	Dmel r16.19	Amel 4.5	Ptep 2.0
Complete	1058 (99.3%)	1061 (99.6%)	1063 (99.8%)	1043 (97.9%)	1007 (94.4%)
Complete single copy	1054 (98.9%)	1056 (99.1%)	1055 (99%)	1038 (97.4%)	966 (90.6%)
Complete duplicated	4 (0.4%)	5 (0.5%)	8 (0.8%)	5 (0.5%)	41 (3.8%)
Fragmented	5 (0.5%)	2 (0.2%)	0 (0%)	15 (1.4%)	18 (1.7%)
Missing	3 (0.2%)	3 (0.2%)	3 (0.2%)	8 (0.7%)	41 (3.9%)
Genes in BUSCO profile	1066	1066	1066	1066	1066

### Official gene set and NCBI RefSeq genome

The genome assembly as well as the gene models have been submitted to Genbank (NCBI) as the RefSeq genome (GCF_000002335.3) and Tribolium (OGS3) (GCA_000002335.3) [48]. Genome assembly 5.2 and gene set OGS3 are available on the NCBI website (ftp://ftp.ncbi.nlm.nih.gov/genomes/all/GCF/000/002/335/GCF_000002335.3_Tcas5.2) and are available as a preselection in several NCBI services, such as the BLAST search.

### Protein sequence conservation

*Drosophila melanogaster* and *Caenorhabditis elegans* are the main invertebrate models for functional genetics and have contributed tremendously to the understanding of cellular and molecular processes relevant for vertebrate biology. However, their protein sequences are quite diverged compared to *Apis mellifera* or the annelid *Platynereis dumerilii* [49]. The transferability of findings to other taxa may depend, among other things, on the biochemical conservation of the proteins involved. Hence, when choosing a model system, the conservation of the proteome is an important aspect. In *Tribolium,* the genetic toolkit is more developed compared to other insects (except for *Drosophila*) or annelids. Unbiased genome-wide screening has been established making *Tribolium* an excellent alternative model for studying basic biological processes. We therefore asked how the protein sequences of the red flour beetle compare to other invertebrate model systems. As outgroup we used the main vertebrate model organism for medical research, the mouse *Mus musculus.*

**Fig. 1:**
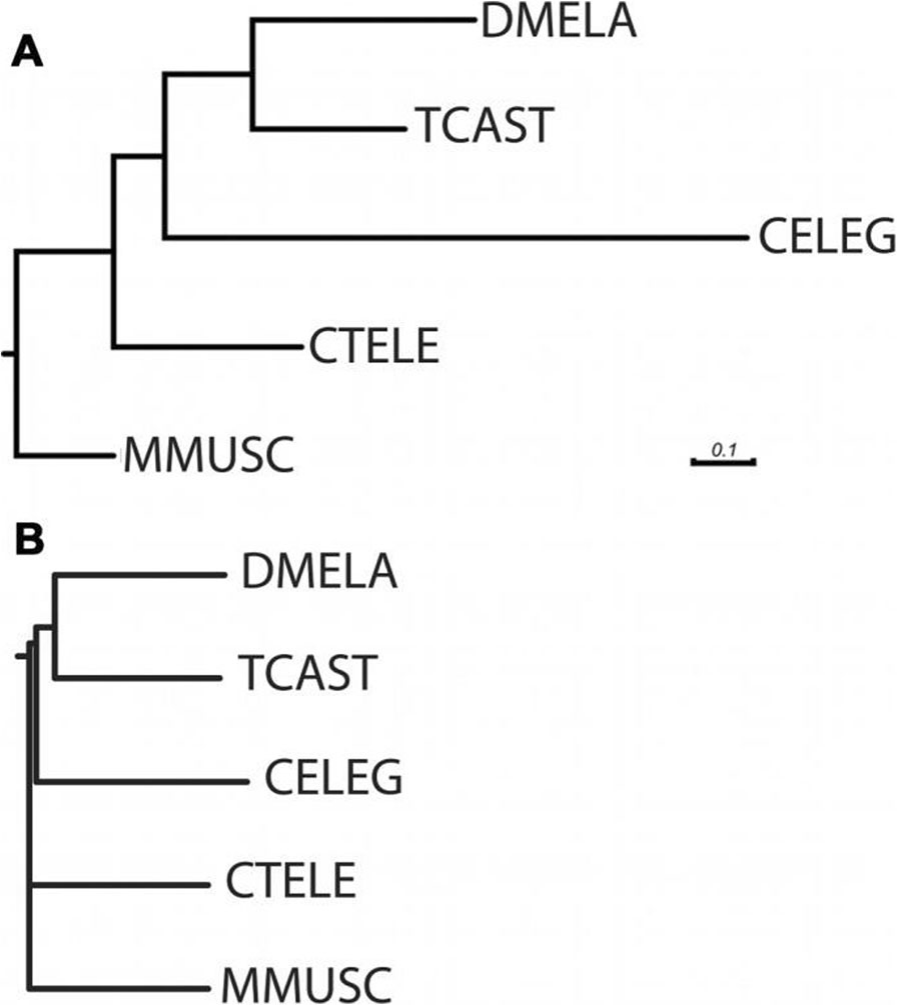
Protein evolution in selected model organisms. **a** An alignment-based comparison of the protein sequences of 1263 single-copy orthologs indicate that the proteome of Tribolium is more conserved than that of the main invertebrate models *Drosophila melanogaster* (DMELA) or *Caenorhabditis elegans* (CELEG). Sequences of annelids are more conserved. Shown is Capitella teleta - see Raible et al. 2005 for *Platynereis dumerilii*. The tree was rooted using the *Mus musculus* (Mammalia) as outgroup. The distances are shown as substitutions per site. **b** An alignment-free comparison shows the same trend but with lower resolution*.* DMELA: *Drosophila melanogaster*; TCAST: *Tribolium castaneum*; CELEG: *Caenorhabditis elegans*; CTELE: Capitella telata; MMUSC: *Mus musculus*

We identified 1263 single-copy orthologs across five species, made an alignment and calculated a phylogenetic tree (Fig. 1a). The *Tribolium* branch is shorter compared to those of *Drosophila* and *C. elegans* indicating that the *Tribolium* proteome is more similar to that of the mouse than are the proteomes of *Drosophila* and *Caenorhabditis.* In this comparison the annelid proteome appears to be even more similar to that of the mouse proteome. In such alignment-based sequence comparisons, the less conserved non-aligneable parts of the proteins are not considered. Therefore, we used an alignment-free method for measuring sequence distances [50, 51] on the same dataset and found it to basically reflect the same conclusion albeit with less resolution (Fig. 1b).

### Prediction of microRNA binding sites

MicroRNAs are short non-coding RNAs that regulate gene expression by guiding the RNA-induced silencing complex (RISC) to complementary sites in the 3’UTR regions of target mRNAs (reviewed in [52]). The principal interaction between microRNAs and their targets occurs through the so-called “seed” region, corresponding to the 2nd and 8th position of the mature microRNA sequence [53], and this complementarity can be used for computational predictions of microRNA-target pairs. Previous studies experimentally identified 347 microRNA genes in the *Tribolium castaneum* genome, each of which can generate two mature microRNAs derived from the two arms (5p and 3p) of the microRNA precursor hairpin (Additional file 1: Table S1) [54, 55] . We extracted the 3’UTR sequences of *Tribolium* protein-coding genes and annotated potential microRNA binding sites in these regions using an algorithm based on the microRNA target recognition principles described in [53]. In addition, we generated an alternative set of computational microRNA target predictions using an algorithm based on the thermodynamic properties of microRNA-mRNA duplexes irrespective of seed complementarity [56]. The two algorithms identified 309,675 and 340,393 unique putative microRNA-target pairs, with approximately 60% overlap. Moreover, a similar number of genes in each set, 13,136 and 13,057 respectively, had at least one microRNA target site.

### Comparison of microRNA target gene sets

MicroRNAs are recognized as important players in animal development, and their role in insects is best understood in the classical model organism *Drosophila melanogaster*. Comparative genomic analyses showed that 83 *Tribolium castaneum* microRNAs have one or more homologs in *Drosophila* [54, 55]. To assess whether conserved microRNAs also have a conserved target repertoire, we sought to assess the number of orthologous genes targeted by each conserved microRNA pair. To this end, we used an identical target prediction approach to determine microRNA-target pairs in *Drosophila melanogaster*, and calculated the numbers of homologous and non-homologous targets for each conserved microRNA pair in the two species (Additional file 1: Table S1). Results indicated that even though the majority of homologous microRNAs have conserved seed sequences for at least one mature product, their target repertoires diverged.

Nonetheless, a subset of well-conserved microRNAs had higher numbers of common predicted targets than expected by chance, especially based on seed complementarity. These included members of the bantam, mir-184, 279/miR-996, mir-2/11/13/2944/6, mir-9, mir-14, mir-1, mir-7, mir-34 seed families, which have been previously identified for their roles in key developmental processes in *Drosophila*, and are highly expressed in both fruit fly and beetle embryos.

Given the large number of target predictions identified for individual microRNAs we examined the specific conserved targets for one of the microRNAs that both exhibited significant target conservation and had well characterized targets in *Drosophila*. The miR-279/miR-996 family has been extensively characterized for its role in regulating the emergence of CO2 sensing neurons and in circadian rhythms. in *Tribolium,* of the nine characterized targets identified in *Drosophila*, one had no clear ortholog (*upd*), four did not have conserved targeted sequences in their UTRs (*STAT*, *Rho1*, *boss*, and *gcm*), but four targets (*nerfin-1*, *esg*, *ru*, and *neur*) had strongly conserved predicted target sites. microRNA regulation of all these four targets has clear functional importance in these developmental processes and two of them (*nerfin-1* and *esg*) work together as key players in the formation of CO_2_ sensing neurons [57].

In summary, we provide an example where conserved microRNA regulate similar developmental pathways between the two taxa. It will be interesting to determine the degree of conservation of the entire microRNA set. The predicted microRNA binding sites are now available as tracks in the genome browser at iBeetle-Base (https://ibeetle-base.uni-goettingen.de/gb2/gbrowse/tribolium/).

## Discussion

With respect to the toolkit for functional genetics in insects, the red flour beetle *Tribolium castaneum* is second only to *Drosophila melanogaster*. The work described here focused on enhancing genomic resources to support functional genetic work in *Tribolium castaneum*. To that end we increased the contiguity of the genome assembly and generated a significantly improved OGS by adding novel information such as splice variants and microRNA target sites.

In order to close gaps and place more contigs on scaffolds, we added data from long-insert jumping libraries and BioNano Genomics optical mapping. It turned out that the latter contributed much more to enhance the previous assembly based on Sanger sequencing: While the first approach increased the N50 by 20% the BioNano Genomics consensus mapping led to another 3-fold increase of the N50. Hence, data from large single molecules is best suited to overcome the limits of sequencing-based assemblies. Compared to the recently re-sequenced genome assembly of the honey bee [46] our scaffold N50 is significant higher (4753 kb compared to 997 kb). This is also true for the number of placed contigs (2149 compared to 5645). However, compared to *Drosophila,* the most thoroughly sequenced insect genome (contig N50 19,478 kb), our improved assembly still lags behind.

The improved genome assembly and extensive RNA-Seq data provided the basis for an enhanced gene prediction. The BUSCO values indicate a more complete OGS, closer to *Drosophila* than to other emerging model insects. Further, 11% more RNA-Seq reads could be mapped to the gene predictions of OGS3 compared to OGS2, which is a relevant increase e.g. for differential gene expression analyses. The overall number of genes did not increase much. On one hand, 1452 genes without sequence similarity to OGS2 were newly added to the gene set. On the other hand, a similar number of genes from OGS2 is not represented in OGS3. These were mostly very short genes not supported by RNA-Seq data. Hence, most of them were probably false predictions in the former gene set.

Qualitative enhancement includes the detection and annotation of alternative splice variants. Since RNAi is splice variant specific in *Tribolium* [58], this opens the possibility to systematically check for differences in the function of isoforms. Further, the inclusion of UTR regions for many more genes enabled us for the first time to comprehensively map candidate microRNA binding sites to our gene set. Indeed, we have identified a large number of microRNA target sites in orthologs of both *Drosophila* and *Tribolium*. The microRNAs that we identified to have conserved targets belong mostly to microRNA families where obvious loss-of-function phenotypes have previously been characterized in other animals. One example is the miR-279/miR-996 family that share a common seed and have been found to play a key role in Drosophila CO2 sensing neurons and ovarian border cell development [57]. A number of the key microRNA targets identified in *Drosophila*, such as *nerfin*, *escargot*, and *neuralized* were predicted to be targets of *Tribolium* miR-279. This striking example of conservation illustrates that further comparative approaches have the potential to identify conserved regulatory networks involving microRNAs within insects based on the resources provided here. Enhanced coverage with RNA data revealed the transcription start sites of most genes, which helps in the design of genome editing approaches and of transgenic constructs based on endogenous enhancers and promoters [22, 23, 35, 59].

Finally, we show that the proteome of *Tribolium* is less diverged from the vertebrate proteome than that of *Drosophila*, which is an argument for using *Tribolium* as alternative model system when the biochemical function of proteins with relevance to human biology is studied.

## Conclusions

The new genome assembly for *Tribolium castaneum* and the respective gene prediction is available at NCBI as a RefSeq genome and a new official gene set (OGS3). This promotes functional genetics studies with respect to a plethora of topics in *Tribolium,* opens the way for further comparative genomics, e.g. with respect to microRNAs, and positions *Tribolium* as a central model organism within insects.

## Methods

### Genome resequencing and assembly

#### Reference genome files

The *T. castaneum* reference genome assembly (Tcas_3.0, NCBI accession number AAJJ01000000) was downloaded from NCBI. The following 23 contigs, which had been marked by NCBI as contaminants were removed: AAJJ01000455, AAJJ01001129, AAJJ01001336, AAJJ01001886, AAJJ01003084, AAJJ01003125, AAJJ01003874, AAJJ01004029, AAJJ01004493, AAJJ01004617, AAJJ01005150, AAJJ01005727, AAJJ01005755, AAJJ01006305, AAJJ01006331, AAJJ01007110, AAJJ01007612, AAJJ01007893, AAJJ01008452, AAJJ01009546, AAJJ01009593, AAJJ01009648, and AAJJ01009654. In addition, the first 411 nucleotides from AAJJ01009651, and the first 1846 and last 46 nucleotides from AAJJ01005383 were removed after being identified as contaminants. The remaining 8815 contigs (N50 = 43 Kb) had been used to construct the 481 scaffolds (N50 = 975 Kb) included in Tcas 3.0. Information from a genetic recombination map based on molecular markers [39], was used to anchor 176 scaffolds in 10 superscaffolds (often referred to as pseudomolecules or chromosome builds). In Tcas 3.0 these are referred to as ChLGX and ChLG2–10, representing the linkage groups in the recombination map. The remaining 305 scaffolds and 1839 contigs that did not contribute to the superscaffolds were grouped together in Beetlebase (http://beetlebase.org or ftp://ftp.bioinformatics.ksu.edu/pub/BeetleBase/3.0/Tcas_3.0_BeetleBase3.0.agp) (unknown placement).

#### Description of Illumina libraries

The DNA used to construct three long-insert jumping libraries (3, 8, and 20 kb target size) was isolated at the Baylor Human Genome Sequencing Center in 2004 for Sanger-based sequencing. Thus, the source of DNA for these data is the same as for the original reference genome. The insert sizes for the three libraries are 3173 bp, 6775 bp, and 34,825 bp, respectively, with 10–15% standard deviation. Library construction, Illumina sequencing and cleaning were performed by MWGOperon (Europe). For all libraries, reads of minimum length 30 bp and maximum 100 bp were retained after cleaning and removal of the internal spacer. The “_1” files contain the forward reads while the “_2” files contain the reverse reads. Reads lacking the spacer or containing insert sequence only on one side of the spacer were not used. Table 6 lists the number of reads and their length for the jumping libraries.

**Table 6:** Mate pairs jumping library statistics

FastQ	Total reads	Total length
3kb_1	23,677,983	2,120,896,823
3kb_2	23,677,983	2,123,186,604
8kb_1	23,202,365	2,093,651,921
8kb_2	23,202,365	2,096,015,114
20kb_1	12,884,671	1,151,209,160
20kb_2	12,884,671	1,153,515,873

#### Scaffolds linked with atlas-link v0.01

Atlas-Link is a software tool that links and orients scaffolds using mate pair libraries (www.hgsc.bcm.edu/software/atlas-link). Scaffolds in the original assembly (Tcas3.0) were indexed using the IS algorithm in BWA prior to running Atlas-Link on each long insert jumping library with the settings described in Additional file 2. Table 7 shows the improvements that were achieved by Atlas-Link. Scaffold order and placement within Chromosome LG builds was used to validate the Atlas -Link output. We used a value of 300 minimum links, which reproduced most of the original order, linking neighboring scaffolds and adding scaffolds that were unplaced in Tcas_3.0. The output AGP file, was renumbered to reflect the NCBI coordinates. Detailed steps and scripts are provided in Additional file 2

**Table 7:** Number of scaffolds and ungapped length before and after running Atlas-Link

Molecule	Scaffolds before	Ungapped length before	Scaffolds after	Ungapped length after	Unplaced scaffolds added	Unplaced ungapped length added
LG1 = X	13	7,011,684	13	7,071,107	2	59,423
LG2	20	14,013,343	18	14,229,660	2	216,317
LG3	35	27,022,651	29	28,072,007	8	1,049,356
LG4	7	11,540,046	6	11,540,046	–	–
LG5	17	13,832,902	17	14,111,830	3	278,928
LG6	15	8,229,537	12	8,262,430	2	32,893
LG7	18	14,841,431	15	15,084,119	3	242,688
LG8	16	12,760,817	14	12,870,760	1	109,943
LG9	21	14,567,469	21	14,900,846	2	333,377
LG10	14	7,043,942	12	7,070,154	1	26,212
Unplaced multi-contig	305	16,272,476	263	14,079,574		
Unplaced single-contig	1839	4,176,957	1820	4,020,722		
Total	2320	151,313,255	2240	151,313,255		

.

#### Contigs extended and gaps closed with GapFiller v1.10

We used the sequence data from the jumping libraries to fill small gaps in the original assembly. Running GapFiller v1.10 to 20 iterations with strict parameters (detailed parameters, and scripts are provided in Additional file 2).

#### Scaffolds joined using BioNano genomics consensus maps

The genome assembly output from GapFiller was used to generate in silico maps for comparison to BioNano consensus maps and refered to as Tcas5.0 in [41]. Table 8 displays the number, length and N50 of the scaffolds before and after consensus mapping.

**Table 8:** Number of scaffolds, scaffolds’ length, and N50 before and after using BNG consensus maps

Molecule	Scaffolds before	Scaffolds after	Length (Mb) before	Length (Mb) after	N50 (kb) before	N50 (kb) after	Unplaced scaffolds added
LG1 = X	13	4	7.34	8.92	1160.70	7264.05	2
LG2	18	8	14.78	15.034064	1207.76	9314.472	0
LG3	29	18	29.78	31.017975	1409.81	2672.697	3
LG4	6	3	12.11	12.24	2906.70	9484.15	0
LG5	17	7	14.64	15.36	1402.64	4484.65	1
LG6	12	9	9.02	9.25	956.12	2189.88	0
LG7	15	6	15.74	16.48	1333.70	8809.74	0
LG8	14	9	13.66	13.98	1312.85	4002.45	1
LG9	21	10	15.81	16.12	893.90	4920.63	0
LG10	12	11	7.54	8.84	1198.49	1224.30	3
Unplaced	2083	2072	20.33	17.35	150.43	104.32	2
Total	2240	2157	160.74	164.60	1160.70	4002.45	12

### Annotation

The reannotation of the protein-coding genes of *Tribolium castaneum* was done in three main steps: 1) automatic gene prediction based on an unpublished intermediate assembly 4.0 with AUGUSTUS [42] incorporating evidence from multiple sources, 2) merging the gene prediction with the previous official gene set OGS2 [37] and 3) a mapping of the new gene set to assembly 5.2 using liftover [60]. Additionally, manual curation and correction was completed for 399 genes. The RNA-seq reads collected in this project are submitted under Bioproject PRJNA275195 (https://www.ncbi.nlm.nih.gov/bioproject/PRJNA275195).

#### Protein-coding genes

AUGUSTUS is a gene prediction tool based on a hidden Markov model that allows one to incorporate extrinsic evidence such as from RNA-Seq or protein homology. Such extrinsic evidence is summarized in the form of so-called ‘hints’ that are input to AUGUSTUS and that represent mostly soft evidence on the location of exons, introns and other gene features.

RNA-Seq libraries of around 6.66 billion reads from the iBeetle consortium and 9 external contributors constitute the majority of evidence. All reads were aligned against the repeat masked genome assembly 4.0 with GSNAP [61]. Hits were filtered according to three criteria. A hit must reach a minimum identity threshold of 92%. Furthermore, a paired read filter was applied: Reads that are paired must not exceed a genomic distance of 200 Kbp and must be correctly oriented towards each other. Subsequently, reads that could not be unambiguously aligned to a single locus (the identities of the two highest-scoring alignments were within 4% of each other) were discarded in order to avoid false positives such as from pseudogenes.

It is often hard to correctly align spliced reads, especially when they are spliced near the beginning or end of the read. Therefore, an iterative mapping approach was applied. First a set of preliminary introns was generated by using the spliced alignments found by GSNAP and by predicting introns ab initio with AUGUSTUS. Removing sequences of these introns produced partial spliced transcripts to which all reads were aligned a second time. We obtained an improved spliced alignment set with additional spliced alignments via a coordinate change induced by the coordinates of the preliminary introns (http://bioinf.uni-greifswald.de/bioinf/wiki/pmwiki.php?n=IncorporatingRNAseq.GSNAP). From the gaps in the read alignments hints on the location of introns were compiled, including the number of reads that support each intron. Further, from the RNA-Seq genome coverage hints on the location of (parts of) exons were generated.

In addition, evidence from 64,571 expressed sequence tags (ESTs), 19,284 proteins of invertebrates (from uniprot/swissprot database), repetitive regions in the genome detected by RepeatMasker (Smit, AFA, Hubley, R & Green, P. *RepeatMasker Open-4.0*.2013–2015, http://www.repeatmasker.org), 387 published coding genes from NCBI, 69 odorant binding Proteins [62] and 60 “gold standard” sequences that derived from single gene sequence analyses by different groups of the *Tribolium* community. The RNA-Seq reads are available at public databases in the Bioproject PRJNA275195.

#### Integration of the previous gene set

Several analyses indicated that the AUGUSTUS gene set is more accurate. First, a higher number of RNA-seq reads mapped to the OGS3 compared to OGS2. Second, a large portion of genes that are present in OGS3 but not OGS2 were confirmed by additional evidence like blast hit or RNA-seq coverage. Third, most of the genes present in OGS2 but “lost” from OGS3 lacked such additional evidence indicating that they had been false positive annotations of OGS2. However, unclear loci remain, in which the true annotation is yet unknown. In order to introduce some stability in the gene set update we kept the old genes when in doubt whether a newly predicted gene with another structure is indeed a correction of the old gene structure. We address the problem of finding such gene structures by introducing the concept of specifically supported genes. Consider a gene *g*_OGS2_ from the previous gene set and a set of overlapping genes *G*_AUG_ from the AUGUSTUS prediction. *g*_OGS2_ is said to be specifically supported, if it has at least one intron supported by RNA-Seq, that none of the genes in *G*_AUG_ have. Additionally, every supported intron of genes in *G*_AUG_ is also in *g*_OGS2_. In OGS3 we kept all specifically supported OGS2 genes and discarded all AUGUSTUS genes overlapping them.

The set of supported intron candidates was compiled from spliced RNA-Seq reads with a number of restrictions. Each intron candidate had to have a length between 32 and 350,000 bp, all splice sites had to be contain the appropriate sequences and the number of hints supporting a contradicting gene structure had to be at most 9 times higher than the number of hints supporting the intron candidate itself.

Additionally, we kept an OGS2 gene that did not overlap any AUGUSTUS gene, if it had homologs in *Drosophila* or other invertebrates or an annotated function (GO term listed in the Gene Ontology database [63]) or was covered by RNA-Seq reads with FPKM ≥ 0.01 (calculated with eXpress [64]). In total we kept 3087 OGS2 genes and 13,413 AUGUSTUS genes.

#### Liftover from assembly 4.0 to assembly 5.2

After a *Tribolium* community call many genes were manually reviewed and edited based on an intermediate assembly 4.0. To preserve manually curated gene structures, we decided to transfer the new gene set to assembly 5.2. We created an assembly map that assigns each base of assembly 4.0 to a base in the new assembly 5.2, if possible. This map file was used to ‘lift’ above gene set to the updated assembly 5.2 using liftOver taken from the UCSC Genome Toolbox (http://hgdownload.cse.ucsc.edu/admin/exe/linux.x86_64.v287/). 337 genes could not be unambiguously and completely mapped. We applied our annotation pipeline to the new assembly and merged the result with the lifted gene set from the previous assembly. Consequently, we were able to identify gene structures for which the improved assembly allowed a better annotation. The new gene set was complemented by 469 gene structures that could only be predicted based on the new assembly. Furthermore, we corrected 745 of the lifted gene structures according to the concept of specific supported genes as described above.

The standard Viterbi algorithm used in AUGUSTUS predicted 159 transcripts with an in-frame stop codon spliced by an intron. To replace them with alternative gene structures that do not contain in-frame stop codons we ran AUGUSTUS with the option –mea = 1 on the affected regions. MEA is an alternative algorithm that can prohibit spliced in-frame stop codons but needs more computational time. During the GenBank submission process some gene models were revised and seven genes were manually edited or deleted based on suggestions from NCBI.

### Orthology assignment and proteome analyses

Orthologs and paralogs between *T. castaneum* and *D. melanogaster* were found using the OrthoDB database [65] and results were formatted accordingly using custom Perl scripts.

For the phylogenetic analysis, we compared *T. castaneum* (Insecta:Coleoptera) with three other invertebrates; *Drosophila melanogaster* (Insecta:Diptera), *Caenorhabditis elegans* (Nematoda) and *Capitella teleta* (Annelida). The mammalian *Mus musculus* was used as outgroup. More specifically, we used OrthoDB and obtained 1263 single-copy orthologs, in order to perform a phylogenomics analysis with RAxML [66]. Briefly, a multiple sequence alignment was built for each orthologous group separately, using MUSCLE [67]. Then, the resulting alignments were trimmed using trimAl [68] with parameters “-w 3 -gt 0.95 -st 0.01” and concatenated using custom Perl scripts. The concatenated alignment was subsequently used to perform a phylogenomic analysis using RAxML 7.6.6 (PROTGAMMAJTT model of amino acid substitutions) with 100 bootstrap replicates. The final tree was edited with EvolView [69] and InkScape 0.91.

The same set of genes was analyzed separately in an alignment independent approach (see Additional file 2 for details). Two approaches were performed using six distance measures (d1, ..., d6): In the first approach, we used ‘gdist’ to determine the pairwise distances between sequences inside the groups, then ‘phylip neighbor’ to compute corresponding phylogenetic trees, rooted by setting MMUSC as outgroup, and computing the consensus tree using ‘phylip consense’. In the second approach, we concatenated sequences in the groups in random order to form five artificial “whole proteom” sequences (one for each of the species), determined their pairwise distances and computed a phylogenetic tree using ‘phylip neighbor’, again setting the MMUSC sequence as outgroup. To check for robustness of the approach and also the influence of sequence lengths we performed these experiments with different subsets: (1) with all 1263 groups and (2) with a subset of the all groups. The subsets we considered were: (2a) groups with a certain minimum sequence length, (2b) only groups whose sequence lengths differed by at most a certain percentage, and (2c - only for experiment (B)) a random selection of groups (for instance, randomly select 80% of all groups for concatenation). Concatenation experiment (B) produced phylogenies that turned out to be almost immune against changes in order of concatenation and considerably robust against restricting consideration to all groups or subsets of groups concatenation. Best signals where obtained by distance d6, which resulted in the phylogeny displayed in Fig. 1b.

### microRNA prediction

Mature sequences of *T. castaneum* microRNAs (Additional file 1) were retrieved from previous annotations [54, 55], and *D. melanogaster* microRNAs were retrieved from miRBase v21 [70]. *D. melanogaster* transcript 3’UTR sequences were retrieved from Flybase r6.09 [71]. MicroRNA target predictions in the two species were performed using two independent approaches. First, we identified target transcripts having regions complementary to the microRNA 7A1, 7 m8 and 8mer seed sequences as described in [53] using a custom script provided by Antonio Marco [54], and the miRanda and TargetScan algorithms [56, 72], with default parameters. Previously established conserved microRNAs between *T. castaneum* and *D. melanogaster* [54, 55] were used to assess conserved microRNA-target pairs. For microRNAs with more than 1 homolog in the other species, we assessed all possible combinations of homologous pairs. The numbers of conserved microRNA-target interactions (homologous microRNAs targeting homologous genes) were calculated using a custom script. The significance of the conserved target pair numbers was assessed by comparison with the number of orthologous genes obtained by random sampling of equal size without replacement 1000 times.

## Supplementary information

**Additional file 1: Table S1.** Table summarizing microRNA data

**Additional file 2.** Details and scripts used for genome assembly and alignment free phylogenetic tree construction.

## Data Availability

The datasets generated and analyzed during the current study are available in the following repositories: The RefSeq genome assembly 5.2 (GCF_000002335.3) and the official gene set for *Tribolium castaneum* (OGS3) (GCA_000002335.3) are available at Genbank (NCBI). (Genbank: https://www.ncbi.nlm.nih.gov/genome/?term=GCA_000002335.3; ftp download: ftp://ftp.ncbi.nlm.nih.gov/genomes/all/GCF/000/002/335/GCF_000002335.3_Tcas5.2) and at iBeetle-Base: https://ibeetle-base.uni-goettingen.de/help/resources The RNA-Seq reads are available at public databases in the Bioproject PRJNA275195 (https://www.ncbi.nlm.nih.gov/bioproject/PRJNA275195). The data and software underlying the alignment free sequence comparison is found in the following repository https://hdl.handle.net/21.11101/0000-0007-D64E-1. It contains: sequence data of the single-copy orthologs; executables of the used software (along with the source code; a jupyter notebook to execute the analysis we have done and a README file.

## Abbreviations

BLAST: Basic local alignment search tool
BLAT: BLAST like alignment tool
bp: base pairs
BUSCO: Benchmarking Universal Single-Copy Orthologs
CDS: Coding sequence
EST: Expressed sequence tag
LG: Linkage group
Mb: Megabases
mRNA: Messenger RNA
OGS3: Official gene set version 3
RNAi: RNA interference
RNA-Seq: Next generation sequencing of mRNAs
Tcas5.2: Official assembly of genomic sequence of *Tribolium castaneum* version 5.2
UTR: Untranslated region
