# Supplementary material for: Enhanced genome assembly and a new official gene set for Tribolium castaneum
Source: BMC Genomics. 2020 Jan 14;21:47. doi: 10.1186/s12864-019-6394-6 (PMC6961396; doi:10.1186/s12864-019-6394-6)
Supplement: Supplementary file 2 — Additional file 2. Details and scripts used for genome assembly and alignment free phylogenetic tree construction. [file 12864_2019_6394_MOESM2_ESM.docx]

# Supplementary file 2

### A) sam_file_list for Atlas-Link

3kb.merged.sam L3K FR 40 3173 constraint

8kb.merged.sam L8K FR 40 6775 constraint

20kb.merged.sam 203K FR 40 34825 constraint

First column lists the path to the merged library file. Second column lists the identifier for that library. Third column lists the orientation of the reads. Fifth column is the mean distance between the paired reads. NEED THE MEANING OF THE REMAINING COLUMNS (4 and 6) FROM BAYLOR!

### B) atlas.link.configure.file for Atlas-Link

<?xml version="1.0"?>

<procedure>

<step level="1" type="2">

<mate_pair>

<lib_size_limit min="1000" max="3700"></lib_size_limit>

</mate_pair>

<min_links>300</min_links>

<excessive_mate_pairs_for_a_edge_dev_factor>15</excessive_mate_pairs_for_a_edge_dev_factor>

<deviate_factor>5</deviate_factor>

</step>

<step level="2" type="2">

<mate_pair>

<lib_size_limit min="3701" max="10000"></lib_size_limit>

</mate_pair>

<min_links>300</min_links>

<excessive_mate_pairs_for_a_edge_dev_factor>15</excessive_mate_pairs_for_a_edge_dev_factor>

<deviate_factor>5</deviate_factor>

</step>

<step level="3" type="2">

<mate_pair>

<lib_size_limit min="10001" max="35000"></lib_size_limit>

</mate_pair>

<min_links>300</min_links>

<excessive_mate_pairs_for_a_edge_dev_factor>15</excessive_mate_pairs_for_a_edge_dev_factor>

<deviate_factor>5</deviate_factor>

</step>

</procedure>

In this file level is the iteration number, a type of 2 indicates that Atlas-Link runs in superscaffolding or upgrade mode, lib_size_limit defines the minimum and maximum jump length for each library, deviate_factor sets the standard deviation of library size difference allowed in each step of linking, and min_links sets the minimum number of mate pairs to initiate a link between two scaffolds.

### C) make_scaffolds_fa.pl used to combine contigs into scaffolds

#!/usr/bin/perl

use strict;

use warnings;

use Bio::DB::Fasta;

use Bio::Seq;

use Bio::SeqIO;

# usage: ./make_scaffolds_fa.pl contigs.fa scaffolds.agp scaffolds.fa

my $contigs_db = Bio::DB::Fasta->new ($ARGV[0], -reindex => 1, -clean => 1);

open AGP, '<', $ARGV[1] or die "Couldn't open $ARGV[1]: $!";

my @agp = <AGP>;

chomp @agp;

close AGP;

my $scaffolds = Bio::SeqIO->new (-file => ">$ARGV[2]", -format => 'Fasta', -flush => 1);

my ($scaffold_name, $scaffold_seq) = ('', '');

for (@agp) {

# 0 - scaffold name; 5 - contig name; 6 - contig start; 7 - contig stop; 8 - contig orientation

my @line = split /\t/;

if ($line[0] ne $scaffold_name) {

if ($scaffold_name ne '') {

# output the scaffold

my $scaffold = Bio::Seq->new (-id => $scaffold_name, -seq => $scaffold_seq);

$scaffolds->write_seq ($scaffold);

}

$scaffold_name = $line[0];

$scaffold_seq = '';

}

if ($line[4] eq 'N') {

$scaffold_seq .= 'N' x $line[5];

}

else {

my $contig = $contigs_db->get_Seq_by_id ($line[5]);

if ($line[8] eq '+') {

$scaffold_seq .= $contig->subseq ($line[6], $line[7]);

}

else {

$scaffold_seq .= $contig->trunc ($line[6], $line[7])->revcom->seq;

}

}

}

# output the last scaffold

my $scaffold = Bio::Seq->new (-id => $scaffold_name, -seq => $scaffold_seq);

$scaffolds->write_seq ($scaffold);

### D) libraries.txt for GapFiller

L3K bwa L3K1.fastq L3K2.fastq 3173 0.75 FR

L8K bwa L8K1.fastq L8K2.fastq 6775 0.75 FR

L20K bwa L20K1.fastq L20K2.fastq 34825 0.75 FR

First column contains the name of the library. Second column indicates the name of the aligner. Third and fourth columns are the names of the files for the paired reads. Fifth column is the mean distance between the paired reads. Sixth column is the deviation of the mean distance that is allowed. Seventh column indicates the orientation of the paired-reads.

### E) fasta_to_agp.pl used to split scaffolds into contigs and generate AGP file

#!/usr/bin/perl

use strict;

use warnings;

use Bio::DB::Fasta;

use Bio::Seq;

use Bio::SeqIO;

# usage: ./fasta_to_agp.pl scaffolds.fa min_gap_length contig_name_prefix scaffolds.agp contigs.fa

# e.g., ./fasta_to_agp.pl gap_filler.fa 10 tcas scaffolds.agp contigs.fa

# e.g., ./fasta_to_agp.pl gap_filler.fa 10 tcas scaffolds.agp

my ($contig_id_suffix) = 1;

my $scaffolds_db = Bio::DB::Fasta->new ($ARGV[0], -reindex => 1, -clean => 1);

my ($min_gap_length, $prefix, $fa) = ($ARGV[1], $ARGV[2], $ARGV[4]);

my $contigs_fa = Bio::SeqIO->new (-file => ">$fa", -format => 'Fasta', -flush => 1) if (defined $fa);

# get scaffold ids in the order they are in the fasta file

my @scaffold_ids;

open FA, '<', $ARGV[0] or die "Couldn't open $ARGV[0]: $!";

while (<FA>) {

if (/^>/) {

chomp;

s/^>//;

push (@scaffold_ids, $_);

}

}

close FA;

# split the scaffolds into contigs and generate the AGP

open AGP, '>', $ARGV[3] or die "Couldn't open $ARGV[3]: $!";

for my $object (@scaffold_ids) {

my ($object_beg, $object_end, $part_number) = (1, 1, 1);

my $scaffold = $scaffolds_db->get_Seq_by_id ($object);

my $object_len_fa = length ($scaffold->seq);

my $scaffold_seq = $scaffold->seq;

while ($scaffold_seq) {

if ($scaffold_seq =~ /N{$min_gap_length,}/) { # there is at least one gap present in the scaffold

my ($contig, $gap) = ($`, $&);

$scaffold_seq = $';

# contig

# fasta

if (defined $fa) {

my $contig_seq = Bio::Seq->new (-id => "${prefix}_$contig_id_suffix", -seq => $contig);

$contigs_fa->write_seq ($contig_seq);

}

# agp

$object_end = $object_beg + length ($contig) - 1;

print AGP "$object\t$object_beg\t$object_end\t", $part_number++, "\tW\t$prefix", "_", $contig_id_suffix++, "\t1\t", length ($contig), "\t+\n";

$object_beg = $object_end + 1;

# gap

$object_end = $object_beg + length ($gap) - 1;

print AGP "$object\t$object_beg\t$object_end\t", $part_number++, "\tN\t", length ($gap), "\tscaffold\tno\tna\n";

$object_beg = $object_end + 1;

}

else { # there are no other gaps in the scaffold

my $contig = $scaffold_seq;

$scaffold_seq = '';

# contig

# fasta

if (defined $fa) {

my $contig_seq = Bio::Seq->new (-id => "${prefix}_$contig_id_suffix", -seq => $contig);

$contigs_fa->write_seq ($contig_seq);

}

# agp

$object_end = $object_beg + length ($contig) - 1;

print AGP "$object\t$object_beg\t$object_end\t", $part_number++, "\tW\t$prefix", "_", $contig_id_suffix++, "\t1\t", length ($contig), "\t+\n";

}

}

}

close AGP;

### F) Alignment free sequence comparison on single-copy orthologs from T.castanea resequencing data

#### Accompanying data+software

The repository with data and software to reproduce all our findings can be accessed via the persistent URL <https://hdl.handle.net/21.11101/0000-0007-D64E-1>. It contains:

- sequence data of the single-copy orthologs
- executables of the used software (along with the source code)
- a jupyter notebook to execute the analysis we have done

along with a README containing detailed description of the material and it's usage as well as (in the jupyter notebook) the log of an example run.

#### Alignment-free phylogenetic analysis

We used distance based methodes as described in [1] to construct phylogenetic trees for single-copy orthologs. Distances (= dissimilarities between sequences) are determined using ideas from information theory (see [2,3]). Appealing variants of this approach have been applied for whole genome phylogenies in [4,5]. We implemented both approaches.

High-level description of the approach from [4] using an example from that paper (based on nucleotides but immediately generalizes to amino acids): The main underlying tool is the so-called LZ-complexity c(S) of a sequence S, like S = AACGTACCATTGACGGTCACCAA. It can be found by reading S from left to right and splitting it after every substring that did not occour earlier. The resulting fragments are A·AC·G·T·ACC·AT·TG·ACGG·TC·ACCAA and the complexity c(S) = 10 is their number. The fragment list is kind of a database that can be used to describe the whole string: E.g., ACCAA (position 10) can be described by referring to earlier seen fragments 5 (once) and 1 (twice). Fragments tend to increase in length and in the long run we will have seen almost every essential substring which could be used to data compress the string: The better compressible c(S) is, the lower c(S) will be.

To define a dissimilarity between sequences S and T, observe that the read-and-split process applied to the concatenation ST can reuse the database for S (we skip some subtle details here) and c(ST) - c(S) measures how much S's database is helpful for compressing also T. Similarily, c(TS) - c(T) measures how much T helps to compress S. The symmetric version d3(S,T) = (c(ST) - c(S)) + (c(TS) - c(T)) is a measure for dissimilarity between S and T and can be used for distance based phylogeny as described above. [4] describes 5 different dissimilarities, all based on LZ-complexity c(·). For example, to account for the influence of sequence length, a normalized version of d3 is d5(S,T) = d3(S,T) / (c(ST) + c(TS)). Somewhat related in flavour but more sophisticated is distance measure d6 from [5]. Furthermore, d6 is said to yield somewhat better signals than d1, …, d5 and our results support that. We implemented all these distance measures in a standalone tool gdist. Then, using tools from the PHYLIP software suite [6], we computed phylogenetic trees.

#### Data and basic experimental setup

Within the resequencing project 1263 single-copy orthologs among *Mus musculus* (as outgroup), *Caenorhabditis elegans, Tribolium castaneum, Drosophila melanogaster* and one Annelida species where identified. These are represented by 5 fasta-files in each group (one for each of TCAST, DMELA, CTELE, MMUSC, CELEG). We analysed these groups with two basic approaches, that we performed separately for each of the implemented distance measures d1, …, d6:

1. (A)
   1. using gdist determine pairwise distances between sequences inside the groups, then using phylip neighbor compute corresponding phylogenetic trees, rooted by outgroup MMUSC. From the resulting 1263 trees we finally compute the consensus tree using phylip consense.
2. (B)
   1. concatenate sequences from the groups in random order to form 5 artificial "whole proteom" sequences (one for each of the species under consideration), determine their pairwise distances and compute a phylogenetic tree from these using phylip neighbor, again setting the (now artificial) MMUSC sequence as outgroup.

To check robustness of the approach and also the influence of sequence lengths we performed these experiments both (1) with all 1263 groups and (2) with subsets of all groups. The subsets we considered where: (2a) groups with a certain minimum sequence length, (2b) only groups whose sequence lengths differ by at most a certain percentage, and (2c - only for experiment (B)) a random selection of all groups (like for instance, randomly select 80% of all groups for concatenation).

#### Results

- Consensus experiment (A) produced the following majority rule consensus tree:

+-------DMELA.fast

+-------|

+-------| +-------TCAST.fast

| |

| +---------------CELEG.fast

|

+-----------------------MMUSC.fast

|

+-----------------------CTELE.fast

In 55%-79% of the trees (DMELA,TCAST) are closest and in 60-85% (DMELA,TCAST,CELEG) are grouped together — figures depend on underlying distance d1, …, d6 and on chosen subset (1, 2a, 2b): Signals tend to get stronger if consideration is restricted to long sequences (e.g., at least 1000) or to comparable lengths sequences (e.g., lengths differing by at most 10%). d1-based signals are much weaker than those based on d2, .., d6, who all yield comparable performance (in terms of partition ratios).

- Concatenation experiment (B) produced phylogenies that turned out to be almost immune against changes in order of concatenation and considerably robust against restricting consideration to all or subset of groups concatenation (1, 2a, 2b, 2c). All of d2, …, d6 partition ((DMELA,TCAST),CELEG) vs. others, regardless of the subset. In general, signals from concatenated sequences get better if restricted to subsets (2a, 2b) and get worse if restricted to a random selection af all groups (2c). Throughout best signals where obtained by distance d6, which in setup (1) resulted in a phylogeny like for example:

+---------------------------------------rCTELE.fas

!

! +------------------------------------rTCAST.fas

! +---3

2-1 +-------------------------------------rDMELA.fas

! !

! +----------------------------------------------rCELEG.fas

!

+---------------------------------------rMMUSC.fas

#### References

- 1. [1]Joseph Felsenstein: Inferring Phylogenies. Sinauer, 2003.
  2. [2]Ming Li, Xin Chen, Xin Li, Bin Ma, Paul M. B. Vitányi: The similarity metric. IEEE Trans. Information Theory 50(12): 3250-3264 (2004)
  3. [3]Rudi Cilibrasi, Paul M. B. Vitányi: Clustering by compression. IEEE Trans. Information Theory 51(4): 1523-1545 (2005)
  4. [4]Hasan H. Otu, Khalid Sayood: A new sequence distance measure for phylogenetic tree construction. Bioinformatics 19(16): 2122-2130 (2003)
  5. [5]Igor Ulitsky, David Burstein, Tamir Tuller, Benny Chor: The Average Common Substring Approach to Phylogenomic Reconstruction. Journal of Computational Biology 13(2): 336-350 (2006)
  6. [6]PHYLIP software suite: <http://evolution.genetics.washington.edu/phylip.html>
